# Pulsed field versus cryoballoon ablation for atrial fibrillation: a real-world observational study on procedural outcomes and efficacy

**DOI:** 10.1007/s12471-023-01850-8

**Published:** 2024-01-30

**Authors:** Mileen R. D. van de Kar, Stacey R. Slingerland, Gijs J. van Steenbergen, Tim Brouwer, Daniela N. Schulz, Dennis van Veghel, Lukas Dekker

**Affiliations:** 1 Catharina Heart Centre, Catharina Hospital, Eindhoven, The Netherlands; 2 Department of Biomedical Technology, Eindhoven University of Technology, Eindhoven, The Netherlands

**Keywords:** Atrial fibrillation, Pulsed-field ablation, Cryoballoon ablation, Real-world data

## Abstract

**Introduction:**

Atrial fibrillation often necessitates catheter ablation when antiarrhythmic drug therapy fails. Single-shot technologies using thermal energy, such as cryoballoon ablation, are commonly used, but pulsed field ablation (PFA), an innovative non-thermal ablation technique, is a potential alternative. This retrospective observational study aimed to compare the safety and efficacy of cryoballoon ablation and PFA in patients undergoing their first pulmonary vein isolation (PVI) procedure for atrial fibrillation treatment.

**Methods:**

We utilised real-world data from patients who underwent PVI using cryoballoon ablation or PFA. The primary outcome encompassed procedural complications, including phrenic nerve palsy, cardiac tamponade, thromboembolic complications, bleeding complications and mortality. Secondary outcomes were procedural characteristics including procedure duration, length of hospital admission, and re-do ablation rates within 6 months.

**Results:**

A total of 1714 procedures were analysed: 1241 in the cryoballoon group and 473 in the PFA group. Gender distribution (*p* = 0.03) and estimated glomerular filtration rate (*p* = 0.01) differed significantly. With regard to the primary outcome, the cryoballoon group demonstrated a higher incidence of phrenic nerve palsy compared with the PFA group (15 vs 0; *p* = 0.02). The procedure duration was shorter in the PFA group, even after adjusting for baseline characteristics (95.0 vs 74.0 min; *p* < 0.001). After adjustment for baseline characteristics, admission duration differed between the groups as well (*p* = 0.04).

**Conclusion:**

The study results supported the safety and efficacy of PFA over cryoballoon ablation for PVI, highlighting advantages such as shorter procedure duration and absence of phrenic nerve palsy.

**Supplementary Information:**

The online version of this article (10.1007/s12471-023-01850-8) contains supplementary material, which is available to authorized users.

## What’s new?

- This retrospective cohort study conducted at a Dutch, high-volume centre compared cryoballoon ablation and pulsed field ablation (PFA) techniques for pulmonary vein isolation (PVI) in patients with atrial fibrillation (AF) using real-world data.
- PFA was associated with a lower incidence of phrenic nerve palsies compared with cryoballoon ablation, indicating its feasibility and potential as a viable alternative technique for PVI in AF patients.
- PFA offered a time-saving advantage over cryoballoon ablation, with a shorter procedure duration, which could improve the procedural efficiency and resource utilisation in clinical practice.

## Introduction

Atrial fibrillation is associated with substantial morbidity and mortality, causing a significant burden to patients, societal health and health economy [1, 2]. AF can be effectively treated with catheter ablation by pulmonary vein isolation (PVI) which has shown to be more effective than medical therapy in reducing hospitalisations and improving quality of life in patients with symptomatic paroxysmal or persistent AF [3–5].

Over the years, 2 dominant ablation techniques using thermal energy have been employed to produce durable lesions at the PV antra: point-by-point (PBP) ablation and single-shot ablation. PBP ablation involves the application of single ablation lesions using a single-tip electrode catheter to produce a contiguous line of transmural scar tissue around the PVs, whereas single-shot ablation employs a multipolar radiofrequency catheter or freezing balloon (cryoballoon), which is placed in the antrum of each individual PV [6]. These advancements have resulted in greater efficacy and efficiency of PVI procedures, especially in the case of cryoballoon ablation [1, 7, 8]. However, both techniques can be challenging, which sometimes leads to incomplete PVI or excessive ablation with thermal damage to surrounding structures and subsequently PV stenosis, phrenic nerve palsy or oesophageal fistula [9].

For the treatment of patients with symptomatic paroxysmal or persistent AF, a novel ablation technology called pulsed field ablation (PFA) has been introduced. The PFA systems FARAPULSE^TM^ and CENTAURI^TM^ utilise both multi-electrode and single-tip catheters, thereby offering a nonthermal ablative technology [3, 10]. As PFA has higher specificity for myocardial cells and the ability to create transmural adjacent lesions through irreversible electroporation, it could provide safety and efficacy advantages over thermal energy ablation [11, 12]. The myocardial specificity potentially limits collateral damage of non-target tissue such as neighbouring nervous or arterial structures or the oesophagus [3, 14–16].

Earlier studies that compared PFA with previous ablation techniques were mostly feasibility trials and observational studies, while randomised controlled trials are limited [11–14]. To ensure the safety of this high-volume PFA technology and optimise catheterisation laboratory planning predictability, it is essential to compare the various techniques. We examined real-world procedural data and short-term clinical outcomes of cryoballoon ablation and multi-electrode PFA performed in a single high-volume centre in the Netherlands.

## Methods

### Study design and population

This retrospective observational study was conducted at the Catharina Hospital Heart Centre in Eindhoven, the Netherlands and ran from 1 January 2019 through 31 December 2022. Cryoballoon ablation was the primary treatment for paroxysmal and persistent AF until January 2022, at which point PFA became the preferred therapy for all AF patients. Consequently, patients were categorised into cryoballoon and PFA groups. Eligible participants were adults aged ≥ 18 years who had undergone either cryoballoon or PFA ablation as their initial AF treatment. Only the first PVI performed during the specified study period was included in the analysis.

### Primary pulmonary vein ablation technique

During the time cryoballoon ablation was used at our hospital, groin puncture was performed blindly or under ultrasound guidance, whereas ultrasound-guided puncture became the standard approach with the implementation of PFA. Cryoballoon ablation utilised 12-French transseptal sheaths, while PFA employed 13-French sheaths. Both procedures involved inserting a second 6‑French sheath for a non-steerable diagnostic catheter, without performing arterial punctures. For cryoballoon ablation, the 27-mm Arctic Front^TM^ catheter (Medtronic, Fridley, MN, USA) was used, and for PFA, the FARAPULSE^TM^ catheter with a size of 31 or 35 mm (Boston Scientific, Marlborough, MA, USA) was employed at the physician’s discretion.

During cryoballoon ablation, one application was administered per PV, each lasting 240 s, unless early termination was deemed necessary due to phrenic capture loss. The application was halted if a temperature of −40 °C was not reached within 60 s, and the balloon position was adjusted accordingly. PV isolation was assessed by placing the diagnostic catheter behind the balloon and evaluating PV signals. The right PVs were ablated with concurrent right phrenic nerve stimulations via subclavian vein and manual diaphragm excursion assessments.

For PV isolation in PFA, a standard of 8 applications was used per PV: 4 in the basket position and 4 in the flower position. Variation in the number of applications was permitted at the physician’s discretion. Verification of PV isolation involved PV stimulation to confirm the absence of PV potentials and the presence of exit block.

Pre-procedural computed tomography scans were not conducted to assess PV anatomy in either group. PV anatomy assessment relied on visual inspection and contrast injection during cryoballoon ablation, with segmented ablation for suspected common ostium cases to prevent excessive depth or ostial lesions. In PFA, circumferential ablation was attempted in suspected cases.

Heparin was administered to maintain an activated clotting time > 300 s, followed by protamine administration, removal of venous sheaths and achievement of haemostasis with manual pressure and a pressure bandage. Patients had a 6-hour bed rest period and were allowed to mobilise for an additional hour before potential same-day discharge if stable and if the procedure finished before 15:00 h.

The follow-up protocol was similar for both ablation techniques. Amiodarone was discontinued immediately after the procedure, while other antiarrhythmic drugs were typically stopped after 3 months of follow-up, unless clinical indications required their continuation.

### Baseline patient and procedural characteristics

Relevant data were extracted from electronic medical records. Based on the impact of outcomes after PVI according to a literature study [17–19], the following baseline characteristics were included: age, gender, body mass index, renal function based on the estimated glomerular filtration rate (eGFR), left ventricular ejection fraction, left atrial volume index (LAVI) and AF type (paroxysmal or persistent).

Procedural measures include energy source/PVI method (cryoballoon or PFA), procedure duration (indicating time between arrival at and departure from procedure room) and admission duration. Baseline and procedural characteristics were defined using the definitions of the Netherlands Heart Registry [20], which align with the European Society of Cardiology and the American College of Cardiology/American Heart Association guidelines [1, 21].

### Primary and secondary outcomes

The primary outcome encompassed procedural complications, specifically phrenic nerve palsy during admission, cardiac tamponade ≤ 30 days, thromboembolic complications ≤ 72 h, bleeding complications during admission and 30-day mortality. Phrenic nerve palsy was defined as persistent phrenic nerve failure (lasting ≥ 24 h) following intervention during admission. Phrenic nerve function was monitored by manual assessment of diaphragm excursions during procedures. Cardiac tamponade ≤ 30 days was characterised by the presence of pericardial fluid volume > 5 mm, with or without clinical or echocardiographic signs necessitating intervention. Thromboembolic complications encompassed thromboembolic events including ischaemic cerebrovascular accident, peripheral emboli and pulmonary emboli occurring ≤ 72 h post-intervention. Bleeding complications comprised groin bleeding requiring surgical intervention or transfusion during admission for the current procedure.

Secondary outcomes involved comparing the overall procedural characteristics of the PFA and cryoballoon ablation techniques, including procedure duration, admission duration, and re-do ablation rates within 6 months. Re-dos included procedures performed and scheduled within 6 months. In both ablation groups, the decision to conduct re-do procedures was primarily symptom-driven.

### Statistical analysis

We assessed the dataset structure and distribution using descriptive statistics, Kolmogorov–Smirnov tests and Q‑Q plots. Continuous variables are presented as mean with standard deviation (SD) for normally distributed variables and as median with interquartile range (IQR) for non-normally distributed variables. Categorical variables are expressed as absolute frequencies (*n*) and relative frequencies. The Mann-Whitney U test, Student’s *t*-test and chi-square test were employed to identify significant differences between the treatment groups, as appropriate.

Multiple logistic regression analyses, adjusted for baseline characteristics, were used to examine the associations between short-term clinical outcomes and the PVI method used (cryoballoon or PFA). Multiple linear regression analyses, also adjusted for baseline characteristics, were utilised to investigate the associations between procedure and admission durations and the PVI methods. Baseline characteristics were included in regression models if the percentage of missing values was < 10%. Statistical significance was set at a two-tailed *p*-value (α < 0.05). SPSS version 29 (IBM, Chicago, IL, USA) was used to perform all data analyses.

## Results

A total of 1714 procedures were included: 1241 in the cryoballoon group and 473 in the PFA group. Among the patients treated with cryoballoon ablation, 859 were males (69.2%), whereas the PFA group comprised 301 men (63.6%; *p* = 0.04) (Tab. 1). The median eGFR was 73.0 ml/min (IQR: 63.0–84.0) in the total study population, but there was a significant difference between the cryoballoon group (74.0 ml/min; IQR: 64.0–84.0) and the PFA group (71.0 ml/min; IQR: 61.0–83.0; *p* = 0.01). The median LAVI of the entire study cohort (*n* = 1042) was 35.5 ml/m^2^ (IQR: 28.8–43.0), with the cryoballoon ablation (*n* = 691) and PFA groups (*n* = 351) showing a significant difference (36.0 ml/m^2^; IQR: 29.0–44.0 vs 35.0 ml/m^2^; IQR: 28.0–42.0; *p* = 0.04). Other baseline variables did not statistically differ (Tab. 1).

**Table 1:** Baseline patient and procedural characteristics

Characteristic	Total cohort (*N* = 1714)	Cryoballoon (*n* = 1241)	PFA (*n* = 473)	*P*-value
Age, years	64 (57–70)	64 (57–70)	65 (58–71)	0.59
Male	1161 (67.7)	859 (69.2)	301 (63.6)	0.04
BMI, kg/m^2^	26.8 (24.6–29.6)	26.8 (24.6–29.7)	26.8 (24.4–29.5)	0.40
eGFR, ml/min	73.0 (63.0–84.0)	74.0 (64.0–84.0)	71.0 (61.0–83.0)	0.01
DM	134 (7.8)	90 (7.3)	44 (9.3)	0.16
LVEF, %	55.0 (55.0–55.0)	55.0 (55.0–55.0)	55.0 (55.0–55.0)	0.59
LAVI, ml/m^2^*	35.5 (28.8–43.0)	36.0 (29.0–44.0)	35.0 (28.0–42.0)	0.04
*AF type*				0.07
– Paroxysmal	1107 (64.6)	818 (65.9)	289 (61.1)	
– Persistent	604 (35.3)	422 (34.0)	182 (38.5)	
– Unknown	3 (0.2)	1 (0.1)	2 (0.4)	

Data are median (interquartile range) or *n* (%)

*BMI* body mass index, *eGFR* estimated glomerular filtration rate, *DM* diabetes mellitus, *LVEF* left ventricular ejection fraction, *LAVI* left atrial volume index, *AF* atrial fibrillation

*Total cohort: *n* = 1042; cryoballoon group: *n* = 691; pulsed field ablation (*PFA*): *n* = 351

Phrenic nerve palsy occurred in 15 cases of the total cohort (0.9%) (Tab. 2). Of these cases, 9 were temporary, with a mean recovery time of 90.7 days (SD: 24.6). Phrenic nerve palsy was only seen in patients receiving cryoballoon ablation and not in the PFA group (*p* = 0.02). Cardiac tamponade was observed in 7 cases, for which a pericardial drain was placed for 24 h. In 3 cases, an antidote to reverse anticoagulation was administered. Cardiac tamponade occurrences were attributed to left atrial perforation during transseptal passage and repositioning manoeuvres of the wire and ablation catheter. No significant differences in other outcomes were observed.

**Table 2:** Univariate analyses of procedural outcomes

Outcome	Total cohort (*N* = 1714)	Cryoballoon (*n* = 1241)	PFA (*n* = 473)	*P*-value
*Primary outcomes*				
– Bleeding complication during admission	16 (0.9)	15 (1.2)	1 (0.2)	0.09
– Phrenic nerve palsy during admission	15 (0.9)	15 (1.2)	0	0.02
– Thromboembolic complication ≤ 72 h	4 (0.2)	3 (0.2)	1 (0.2)	1.00
– Cardiac tamponade ≤ 30 days	7 (0.4)	4 (0.3)	3 (0.6)	0.40
– Mortality ≤ 30 days	1 (0.1)	1 (0.1)	0	0.53
*Secondary outcomes*				
– Procedure duration, min	91.0 (78.8–105.0)	95.0 (85.0–108.0)	74.0 (65.0–87.5)	< 0.001
– Admission duration, days	0 (0–1)	0 (0–1)	0 (0–1)	0.12
– Re-do ablation ≤ 6 months	159/1638 (9.7)	121 (9.8)	38/397 (9.6)	0.92

Data are *n* (%) or median (interquartile range)

*PFA* pulsed field ablation

The median procedure duration of the total cohort was 91.0 min (IQR: 78.8–105.0) (Tab. 2). The median duration was longer in the cryoballoon group (95.0 min; IQR: 85.0–108.0) and shorter in the PFA group (74.0 min; IQR: 65.0–87.5; *p* < 0.001). Re-do rates within 6 months were similar in both groups (9.8% for cryoballoon ablation vs 9.6% for PFA).

After adjustment for baseline characteristics, the PVI techniques showed significant differences in procedure duration (*p* < 0.001) and admission duration (*p* = 0.04) (Tab. 3, see also Tables S1 and S2 in Electronic Supplementary Material). Due to the absence of phrenic nerve palsies and mortality cases in the PFA group, logistic regression analysis could not be performed for these outcomes.

**Table 3:** Multivariate logistic and linear regression analyses of correlation between PVI technique and outcomes

Outcome	OR/beta	95% CI	*P*-value
*Primary outcomes*			
– Bleeding complication during admission	0.16	0.02–1.25	0.08
– Phrenic nerve palsy during admission	0	0	NA
– Thromboembolic complication ≤ 72 h	0.83	0.08–8.08	0.87
– Cardiac tamponade ≤ 30 days	2.07	0.45–9.68	0.36
– Mortality ≤ 30 days	0	0	NA
*Secondary outcomes*			
– Procedure duration	−17.55^a^	−20.05 to −15.03	<0.001
– Admission duration	−0.08^a^	−0.16 to −0.004	0.04
– Re-do ablation ≤ 6 months	1.00	0.68–1.47	0.99

*PVI* pulmonary vein isolation, *OR* odds ratio, *CI* confidence interval, *NA* not applicable

^a^Beta

## Discussion

In this retrospective study, we compared the procedural and short-term clinical outcomes of single-shot cryoballoon ablation and PFA as initial AF ablation techniques at a Dutch, high-volume centre in the Netherlands. Several key findings emerged. First, PFA was associated with a lower occurrence of phrenic nerve palsy than cryoballoon ablation. No significant differences were observed between the 2 techniques in raw and adjusted outcomes, including mortality, vascular complications, thromboembolic events, cardiac tamponade and re-do rates. Additionally, PFA had a shorter duration than cryoballoon ablation, even after accounting for patient characteristics. These findings underscore the potential advantages of PFA, such as reduced phrenic nerve palsy risk, shorter procedure time, possible cost savings and improved patient satisfaction. Notably, these results are consistent with those from a recent study by Urbanek et al. [22], further supporting PFA’s favourable profile compared with cryoballoon ablation.

The higher number of phrenic nerve palsies in the cryoballoon group compared with the PFA group can be attributed to the distinct characteristics of the techniques. Cryoballoon ablation utilises extreme cold temperatures and carries a greater risk of unintended phrenic nerve involvement [8, 9, 23]. In contrast, PFA, which uses non-thermal energy pulses, selectively targets myocardial cells and minimises the risk of damaging nearby structures such as nerves [14–16, 24]. Consequently, the absence of phrenic nerve palsies in the PFA group supports the notion that this technique carries a lower risk of nerve-related complications. In our study cohort, 9 out of 15 phrenic nerve palsies were temporary, a rate that is consistent with that described in the existing literature [25]. These findings highlight the importance of considering potential complications associated with different ablation methods and reinforce the favourable safety profile of PFA in minimising nerve-related adverse events during AF treatment.

While AF recurrence and the need for repeat procedures are well-documented outcomes with cryoballoon ablation, limited data exist on AF recurrence beyond 6 months with PFA. We assessed 6‑month AF re-do rates and found similar results in the cryoballoon (9.8%) and PFA (9.6%) groups, without statistically significant differences. A previous study reported a 1-year freedom rate from atrial arrhythmias after PFA of ~80% [13]. PFA’s potential for more precise lesion creation compared with cryoballoon ablation is supported by the literature [26, 27]. Accurate lesion formation is essential for effective treatment and reduction of recurrent arrhythmias [28].

The literature also highlights lower complication rates of PFA compared with cryoballoon ablation [29]. Although we did not observe a reduction in the number of complications with PFA compared with cryoballoon ablation, except for phrenic nerve palsy, these findings hold promise. The PFA group demonstrated a notably shorter average procedure time of ~20 min, which has the potential for cost reductions. Further studies are needed to investigate the costs or cost-effectiveness of PFA.

### Strengths, limitations and recommendations

This study leveraged real-world observational data to provide insights into the effectiveness and safety of cryoballoon ablation and PFA for AF across diverse patient populations and real-world settings. As of 2022, all patients who have undergone cryoballoon ablation in the past will now receive PFA treatment at our centre, ensuring that there is no selection bias and that significant changes in patient characteristics or referral patterns have not occurred. Therefore, we consider the 2 patient populations as comparable, despite the limitation stemming from the larger patient population in the cryoballoon group, which could impact results by increasing the likelihood of detecting statistically significant differences between groups. While there was a statistically significant difference in LAVI, its clinical relevance may be limited, with suspected non-random missing data warranting cautious interpretation.

It is essential to acknowledge the limitation of assessing AF re-do rates within 6 months as an indicator of AF recurrence, and this should be considered when interpreting our findings. Data on long-term outcomes, such as mortality, complications beyond 1 year and quality of life, were unavailable due to the study’s retrospective nature. Additionally, the retrospective design introduced potential biases, which we addressed by performing multivariate analyses. Still, it cannot be completely ruled out that residual confounding by variables such as mitral valve regurgitation may still be present. The transition to PFA at our hospital also introduced a potential confounding risk by the incorporation of echo-guided groin puncture. Nevertheless, the difference in vascular event rates did not reach statistical significance in this study.

Without pre-procedural imaging, the precise dimensions and morphology of the PVs during the PFA procedure remained uncertain due to the absence of contrast administration. The literature suggests that routine pre-procedural anatomical assessment does not improve clinical outcomes [30]. As a result, this is no longer included in our protocol, aligning with the approach employed in the ADVENT trial (ClincialTrials.gov number: NCT04612244), wherein only left atrial imaging is performed—through transoesophageal or intracardiac echocardiography—to rule out the presence of a left atrial thrombus.

Long-term follow-up and cost-effectiveness assessments are recommended for future research to evaluate the durability, overall impact and cost-effectiveness of PFA. Further studies, such as the ADVENT trial, are needed to confirm these findings and assess the long-term outcomes of PFA in AF treatment.

## Conclusion

This retrospective observational analysis provides evidence that multi-electrode PFA is a safe and effective alternative to cryoballoon ablation for the treatment of AF, with re-do rates assessed within 6 months. PFA offers advantages over cryoballoon ablation in terms of a shorter procedure time and absence of phrenic nerve palsy. These characteristics make PFA a compelling technique for further exploration and development in the field of cardiac ablation, potentially offering improved outcomes for patients with arrhythmias.

### Supplementary Information

**Table S1** Logistic regression analysis

**Table S2** Linear regression analysis

## References

[1] Hindricks G, Potpara T, Dagres N (2020). ESC Guidelines for the diagnosis and management of atrial fibrillation developed in collaboration with the European Association for Cardio-Thoracic Surgery (EACTS): The Task Force for the diagnosis and management of atrial fibrillation of the Europea. Eur Heart J.

[2] Benjamin EJ, Muntner P, Alonso A (2019). Heart Disease and Stroke Statistics—2019 Update: A&nbsp;Report From the American Heart Association. Circulation.

[3] Reddy VY, Neuzil P, Koruth JS (2019). Pulsed Field Ablation for Pulmonary Vein Isolation in Atrial Fibrillation. J Am Coll Cardiol.

[4] le Polain de Waroux J-B, Talajic M, Khairy P (2010). Pulmonary vein isolation for the treatment of atrial fibrillation: past, present and future. Future Cardiol.

[5] Calkins H, Hindricks G (2017). HRS/EHRA/ECAS/APHRS/SOLAECE expert consensus statement on catheter and surgical ablation of atrial fibrillation. Hear Rhythm.

[6] Reddy SA, Nethercott SL, Khialani BV, Virdee MS (2021). Pulmonary vein isolation for atrial fibrillation: Does ablation technique influence outcome?. Indian Heart J.

[7] Packer DL, Kowal RC, Wheelan KR, et al. Cryoballoon ablation of pulmonary veins for paroxysmal atrial fibrillation: first results of the North American Arctic Front (STOP AF) pivotal trial. J Am Coll Cardiol. 2013;61:1713–23. 10.1016/j.jacc.2012.11.064 23500312

[8] Kuck K-H, Brugada J, Fürnkranz A (2016). Cryoballoon or Radiofrequency Ablation for Paroxysmal Atrial Fibrillation. N Engl J Med.

[9] Muthalaly RG (2018). Temporal trends in safety and complication rates of catheter ablation for atrial fibrillation. J Cardiovasc Electrophysiol.

[10] Hartl S, Reinsch N, Füting A, Neven K (2023). Pearls and Pitfalls of Pulsed Field Ablation. Korean Circ J.

[11] Verma A, Boersma L, Haines DE (2022). First-in-Human Experience and Acute Procedural Outcomes Using a&nbsp;Novel Pulsed Field Ablation System: The PULSED AF Pilot Trial. Circ Arrhythm Electrophysiol.

[12] Di Biase L, Diaz JC, Zhang X-D, Romero J (2022). Pulsed field catheter ablation in atrial fibrillation. Trends Cardiovasc Med.

[13] Reddy VY, et al. Pulsed Field Ablation of Paroxysmal Atrial Fibrillation: 1‑Year Outcomes of IMPULSE, PEFCAT, and PEFCAT II. JACC Clin Electrophysiol. 2021;7:614–27. 10.1016/j.jacep.2021.02.014 33933412

[14] Bradley CJ, Haines DE (2020). Pulsed field ablation for pulmonary vein isolation in the treatment of atrial fibrillation. J Cardiovasc Electrophysiol.

[15] Reddy VY, Koruth J, Jais P, et al. Ablation of Atrial Fibrillation With Pulsed Electric Fields: An Ultra-Rapid, Tissue-Selective Modality for Cardiac Ablation. JACC Clin Electrophysiol. 2018;4:987–95. 10.1016/j.jacep.2018.04.005 30139499

[16] Cochet H (2021). Pulsed field ablation selectively spares the oesophagus during pulmonary vein isolation for atrial fibrillation. Europace.

[17] Jaïs P, Cauchemez B, Macle L, et al. Catheter ablation versus antiarrhythmic drugs for atrial fibrillation: the A4 study. Circulation. 2008;118:2498–505. 10.1161/CIRCULATIONAHA.108.772582 19029470

[18] Berruezo A, Tamborero D, Mont L (2007). Pre-procedural predictors of atrial fibrillation recurrence after circumferential pulmonary vein ablation. Eur Heart J.

[19] Lee W-C, Wu P-J, Fang C-Y, Chen H-C, Chen M-C (2021). Impact of chronic kidney disease on atrial fibrillation recurrence following radiofrequency and cryoballoon ablation: A&nbsp;meta-analysis. Int J Clin Pract.

[20] Netherlands Heart Registration. Data dictionary Ablation Registration. http://nederlandsehartregistratie.nl/handboeken/. Published 2020.

[21] January CT, Wann LS, Calkins H (2019). AHA/ACC/HRS Focused Update of the 2014 AHA/ACC/HRS Guideline for the Management of Patients With Atrial Fibrillation: A&nbsp;Report of the American College of Cardiology/American Heart Association Task Force on Clinical Practice Guidelines and the Heart R. Circulation.

[22] Urbanek L (2023). Pulsed Field Versus Cryoballoon Pulmonary Vein Isolation for Atrial Fibrillation: Efficacy, Safety, and Long-Term Follow-Up in a&nbsp;400-Patient Cohort. Circ Arrhythm Electrophysiol.

[23] Sánchez-Quintana D (2009). Anatomic evaluation of the left phrenic nerve relevant to epicardial and endocardial catheter ablation: implications for phrenic nerve injury. Hear Rhythm.

[24] Howard B, Haines DE, Verma A (2022). Characterization of Phrenic Nerve Response to Pulsed Field Ablation. Circ Arrhythm Electrophysiol.

[25] Mol D, Renskers L, Balt JC (2022). Persistent phrenic nerve palsy after atrial fibrillation ablation: Follow-up data from The Netherlands Heart Registration. J Cardiovasc Electrophysiol.

[26] Magni FT, Mulder BA, Groenveld HF (2022). Initial experience with pulsed field ablation for atrial fibrillation. Front Cardiovasc Med.

[27] Kueffer T, Baldinger SH, Servatius H (2022). Validation of a&nbsp;multipolar pulsed-field ablation catheter for endpoint assessment in pulmonary vein isolation procedures. Europace.

[28] Shaheen N, Shaheen A, Ramadan A, Nashwan AJ. Efficacy and safety of novel pulsed field ablation (PFA) technique for atrial fibrillation: A systematic review and meta-analysis. Heal Sci reports. 2023;6:e1079. 10.1002/hsr2.1079 PMC9852677 36698714

[29] Di Monaco A, Vitulano N, Troisi F (2022). Pulsed Field Ablation to Treat Atrial Fibrillation: A&nbsp;Review of the Literature. J Cardiovasc Dev Dis.

[30] Mulder BA (2019). Pulmonary vein anatomy addressed by computed tomography and relation to success of second-generation cryoballoon ablation in paroxysmal atrial fibrillation. Clin Cardiol.

