# Supplementary material for: Pulsed field versus cryoballoon ablation for atrial fibrillation: a real-world observational study on procedural outcomes and efficacy
Source: Neth Heart J. 2024 Jan 30;32(4):167–72. doi: 10.1007/s12471-023-01850-8 (PMC10951164; doi:10.1007/s12471-023-01850-8)
Supplement: Supplementary file 1 — Table S1 Logistic regression analysis [file 12471_2023_1850_MOESM1_ESM.docx]

**Table S1** Logistic regression analysis

|  | **Vascular complication** | | | **Cardiac tamponade** | | | **Mortality** | | | **Thromboembolic complication** | | | **Re-do ablation** | | |
| --- | --- | --- | --- | --- | --- | --- | --- | --- | --- | --- | --- | --- | --- | --- | --- |
|  | **OR** | **95% CI** | **p-value** | **OR** | **95% CI** | **p-value** | **OR** | **95% CI** | **p-value** | **OR** | **95% CI** | **p-value** | **OR** | **95% CI** | **p-value** |
| Ablation method (cryo-balloon) | .16 | .02-1.25 | .08 | 2.07 | .44-9.68 | .36 | 0 | .00-/ | 1.00 | .83 | .09-8.08 | .87 | 1.00 | .68-1.47 | .99 |
| Diabetes Mellitus | .83 | .11-6.61 | .86 | 0 | .00-/ | 1.00 | 1,5E+18 | .00-/ | .91 | 0 | .00-/ | 1.00 | 1.14 | .65-2.03 | .65 |
| Gender (male) | 1.38 | .48-3.96 | .55 | 4.19 | .73-23.99 | .11 | 1,6E+14 | .00-/ | .97 | 1.77 | .22-14.25 | .59 | 1.01 | .69-1.46 | .97 |
| BMI | 1.02 | .90-1.15 | .81 | 1.18 | .99-1.41 | .06 | 0,004 | .00-4.54^E^+102 | .97 | 1.03 | .81-1.31 | .82 | 1.07 | 1.02-1.11 | .00 |
| eGFR | .98 | .95-1.01 | .20 | 1.03 | .99-1.07 | .18 | 0,86 | .00-1.87^E^+18 | .99 | .98 | .92-1.05 | .58 | 1.01 | 1.00-1.02 | .05 |
| LVEF | 1.06 | .96-1.16 | .25 | .96 | .86-1.07 | .48 | 0,13 | .00-6.64^E^28 | .95 | 1.04 | .87-1.24 | .69 | .99 | .97-1.02 | .54 |
| Age | .99 | .94-1.06 | .94 | 1.07 | .97-1.19 | .16 | 0,49 | .00-3.32^E^+55 | .99 | .99 | .89-1.01 | .81 | 1.01 | .99-1.03 | .44 |
| Atrial fibrillation type (paroxysmal) | .44 | .12-1.59 | .21 | 1.15 | .25-5.27 | .86 | 0 | .00-/ | .98 | .64 | .06-6.41 | .70 | 1.56 | 1.10-2.21 | .01 |

** CI = Confidence interval, OR = odds ratio.*

**Vascular complication during admission, cardiac tamponade and mortality within 30 days, thromboembolic complication within 72 hours, re-do ablation within 6 months*
