# Supplementary material for: Pulsed field versus cryoballoon ablation for atrial fibrillation: a real-world observational study on procedural outcomes and efficacy
Source: Neth Heart J. 2024 Jan 30;32(4):167–72. doi: 10.1007/s12471-023-01850-8 (PMC10951164; doi:10.1007/s12471-023-01850-8)
Supplement: Supplementary file 2 — Table S2 Linear regression analysis [file 12471_2023_1850_MOESM2_ESM.docx]

**Table S2** Linear regression analysis

|  | **Procedure duration** | | | **Admission duration** | | |
| --- | --- | --- | --- | --- | --- | --- |
|  | **Beta** | **95% CI** | **p-value** | **Beta** | **95% CI** | **p-value** |
| Ablation method (cryo-balloon) | -.32 | -20.07- -15.03 | .00 | -.05 | -.16- -.004 | .04 |
| Diabetes Mellitus | .03 | -1.43-7.06 | .19 | .05 | -.005-.26 | .06 |
| Gender (male) | .03 | -.93-4.10 | .22 | .03 | -.029-.13 | .22 |
| BMI | -.008 | -.36-.25 | .74 | .008 | -.008-.01 | .76 |
| eGFR | .003 | -.07-.08 | .91 | -.08 | -.006- -.001 | .00 |
| LVEF | -.05 | -.34-.006 | .06 | -.009 | -.006-.004 | .74 |
| Age | -.03 | -.19-.06 | .30 | .02 | -.002-.006 | .39 |
| Atrial fibrillation type (paroxysmal) | .02 | -1.28-3.63 | .35 | -.02 | -.10-.05 | .55 |

**CI = confidence interval.*
